# Investigation of Dental and Oral Health in Children and Adolescents with Special Support Needs from a Child and Adolescent Psychiatric Perspective

**DOI:** 10.3390/children11030355

**Published:** 2024-03-17

**Authors:** Dustin Fornefeld, Oliver Fricke, Andreas G. Schulte, Peter Schmidt

**Affiliations:** 1 Department of Special Care Dentistry, Witten/Herdecke University, Alfred-Herrhausen-Str. 50, 58448 Witten, Germany; andreas.schulte@uni-wh.de (A.G.S.); peter.schmidt@uni-wh.de (P.S.); 2 Department of Child and Adolescent Psychotherapy and Psychiatry, Gemeinschaftskrankenhaus Herdecke, Gerhard-Kienle-Weg 4, 58313 Herdecke, Germany; 3 Department of Human Medicine, Faculty of Health, Witten/Herdecke University, Alfred-Herrhausen-Str. 50, 58488 Witten, Germany; oliver.fricke@uni-wh.de; 4 Department of Child and Adolescent Psychiatry and Psychotherapy, Klinikum Stuttgart, Prießnitzweg 24, 70374 Stuttgart, Germany

**Keywords:** dental health services, special care dentistry, disability evaluation, mental disorders, autism spectrum disorder, oral health

## Abstract

Background: The current literature lacks scientific research on child and adolescent psychiatrists’ (CAPPS) perspectives on dental and oral health. This study aims to investigate the opinions and approaches of child and adolescent psychiatrists and their patients regarding oral and dental health. Methods: A questionnaire-based cross-sectional study was conducted among members of the Professional Association for Child and Adolescent Psychiatry, Psychosomatics and Psychotherapy in Germany. Results: Out of the association members, 10.9% (*n* = 109) participated, with 5.2% (*n* = 52; 38f/14m) completing the questionnaire. Dental and oral health topics were discussed with one-fifth of the patients (19.2%), while 11.5% reported that they were “never” a part of their therapy. Patient-related concerns about dental and oral health were primarily brought into the context of child and adolescent psychiatric work. Dental treatment anxieties were prominent. Only 3.8% of the participants regularly assigned diagnoses related to dental status. The CAPPS employ a bio-psycho-social model for the genesis of oral health-related conditions in Children and Adolescents with Special Needs. Conclusions: CAPPS have a foundation in relationship-based work for assessing oral and dental healthcare and providing recommendations for further dental care. Regional networking and science must be further developed.

## 1. Introduction

At the end of 2021, 7.8 million individuals with severe disabilities were residing in Germany [1]. Of these, 198,325 people (2.5%) were under the age of 18 at the time of data collection. A cerebral, intellectual or mental disability was present in 23% of the recorded individuals with severe disabilities, suggesting that approximately 45,000 children and adolescents could be considered to have a severe disability [1]. In addition, there exists a multitude of mental health conditions, which, although they may result in disability in daily life, are not classified or documented as severe disabilities. The prevalence of mental health disorders among children and adolescents in Germany is estimated to be between 10% and 20%, with a certain degree of underreporting suspected in the realms of both severe disabilities and mental health conditions [2,3,4].

Data from Germany indicate that children and adolescents with disabilities or psychologically induced behavioral disorders exhibit a significantly higher caries prevalence compared to their peers in the general population [5,6,7,8]. Disparities persist into adulthood, where, for instance, individuals with intellectual or physical disabilities are more likely to have missing teeth than their age-matched counterparts in the general population [5]. The evaluation of data concerning 6- to 7-year-olds explicitly notes that this disparity in oral health did not emerge during school years but began prior to school entry [5]. Internationally, across age groups (1–75 years), individuals with intellectual disabilities demonstrate poorer oral health (a higher incidence of conditions such as caries, gingivitis and periodontitis) compared to age-matched peers in the general population [8]. Limited access to (free/affordable) healthcare systems is cited among the multifaceted reasons for this discrepancy [8]. This is pertinent, as the dental and oral health status, along with the presence of dentogenic pain, directly impacts the affected individual’s quality of life [9].

From a dental perspective, patient-related factors such as a lack of cooperation, communication barriers, insufficient general hygiene skills and the need for supportive oral care are chiefly cited for this observation. Moreover, the inadequate inclusion of disability-oriented (dental) medicine in dental education or training is also identified as a reason for the inferior dental care of people with disabilities [10,11]. Numerous data exist on the relationship between the education of dental professionals and their confidence in treating individuals with disabilities (e.g., [12,13,14]).

In addition to dental treatment and care for children and adolescents with intellectual and/or physical disabilities, treating individuals with mental illnesses and neurological developmental disorders is particularly challenging [15,16]. The initial data from Germany show that children and adolescents (6–17 years) undergoing inpatient psychiatric treatment have a higher caries prevalence than their age-matched peers in the general population [17]. This underscores that children and adolescents with disabilities, mental illnesses or neurological developmental disorders constitute a vulnerable patient group experiencing barriers in dental care and treatment. 

In contrast to outpatient child and adolescent psychiatric treatment, where interdisciplinary care in social psychiatric practices and centers is often provided, interdisciplinary care structures are often lacking in dentistry. In summary, in Germany, every dentist may encounter a child or adolescent with intellectual/physical disabilities or mental illnesses requiring treatment, and every child and adolescent psychiatrist and psychotherapist (CAPPS) must anticipate treating individuals with mental illnesses or intellectual/physical disabilities exhibiting untreated or undetected dental and oral conditions. 

### Aim of the Study

A description, analysis or problem indication for child and adolescent psychiatric perspectives—extending beyond the evaluation of concepts for treating dental anxiety, considerations of the transgenerational transmission of dental anxiety and the correlation with socioeconomic factors—was singularly provided in July 1977 [18]. In this context, the expertise and perspective of child and adolescent psychiatry are largely unheard, despite the necessity for specific care concepts for this vulnerable group. It is crucial to understand not only the extent to which dentists are trained for the dental care of patients with intellectual/physical disabilities and mental health issues but also how CAPPS pay attention to aspects of dental and oral health. Furthermore, if necessary, cooperations and networks ensuring that patients receive the best possible dental and medical care and treatment should be established. This article represents, to the best knowledge of the author’s team, for the first time in almost 50 years, data at the intersection of child and adolescent psychiatry and dentistry from Germany.

## 2. Materials and Methods

The present questionnaire-based cross-sectional study was conducted in collaboration with the Professional Association for Child and Adolescent Psychiatry, Psychosomatics and Psychotherapy in Germany (*Berufsverband für Kinder- und Jugendpsychiatrie, Psychosomatik und Psychotherapie in Deutschland e. V*.; abbreviated as BKJPP). The BKJPP was addressed through collaborative efforts of the working group, consisting of experts in the field of child and adolescent psychiatry (OF) and experts in the field of special care dentistry (PS), who were contacted and invited to collaborate. The BKJPP is a national association comprising approximately 1000 CAPPS members (self-disclosure according to the website; accessed on 25 September 2023), and its office is located in the city of Mainz, Germany. This professional association is a German professional association represented in all 16 German federal states, thus embodying explicit national expertise. In Germany, CAPPS must first complete a minimum of six years of medical school and then successfully finish a five-year consecutive specialist training to be qualified as CAPPS and receive the permission to open a practice. CAPPS provide medical care to children and adolescents aged mostly between 0 and 21 years who have a mental disorder or comorbid mental disorder (e.g., according to the ICD-10 F-chapter). They employ psychopharmacological treatment or another approved therapy approach (e.g., cognitive-behavioral therapy) as part of their medical practice. We considered surveying this group of specialists as the most appropriate method for addressing our research question. The surveyed individuals are members of the professional association and thus recognized experts in this field. The BKJPP is primarily committed to improving the care of young people with mental and/or psychosomatic illnesses. To achieve this goal, the BKJPP organizes its own continuing education programs, conferences and congresses, establishes specific research funding and serves as a representative of interests towards politicians and social insurance agencies. Collaboration with other national or international associations has not occurred to date. In terms of participation, fundamental parameters such as the type, conduct and scope were coordinated in advance with the contact person of the professional association. The association did not influence the design, analysis and interpretation of the data.

### 2.1. Survey via Online and Paper Questionnaires 

BKJPP members were invited to participate in the present survey through the internal mailing list. There were two reminders spaced four weeks apart, resulting in a total of three prompts for participation. Ethics approval was not required for this survey, as the anonymized survey of adult healthcare workers does not necessitate ethics approval (see references [11,15]). The study was conducted online using the SoSci platform (SoSci Survey GmbH, Munich, Germany) between 30 May 2022 and 15 November 2022. Additionally, a face-to-face option was provided during the BKJPP conference in 2022 (17 November 2022 to 19 November 2022, Kassel, Germany) to allow association members the opportunity to participate anonymously on-site. The results of the paper questionnaire were manually entered into the database. All data were collected in accordance with the General Data Protection Regulation.

A questionnaire specifically designed for this purpose, with 49 questions, including 39 closed-ended questions and 10 open-ended questions, was utilized. The questionnaire was developed and evaluated in advance by PS, AGS and OF and subsequently digitized for data collection purposes. Following its transfer to the SoSci platform, the correctness and completeness of the questionnaire in digital form were verified, and upon approval by the author group, it was sent to the BKJPP along with the invitation to participate and further information and explanations. 

The questionnaire used in our study has been previously applied in other studies [11] and was validated according to the concept of content validation [19]. It was designed based on existing theories and generated research findings and was then reviewed by experts. Various expert and working groups and feedback guided the questionnaire development process, and assessments from respondents were used to refine it further. The questionnaire was based on similar previous investigations [10,13,16] aiming to enable comparability between different occupational and target group samples in further analysis. 

Participation in the survey lasted approximately 20–30 min and was conducted in German. The participants were informed in advance that participation was voluntary and anonymous. Additionally, they had to confirm their adulthood and indicate that they had read, understood and accepted the consent form. Only fully completed questionnaires were included in the present analysis.

### 2.2. Data Analysis Methods

The collected data were statistically processed using SPSS Version 29 (IBM Corporation, Armonk, NY, USA). Descriptive analyses (e.g., frequency) were conducted. For the question this study aims to address, no validated categories or theories that could be used for quantitative analysis were available. Therefore, it was necessary to employ a theory-driven approach that appropriately evaluates the available material. The Qualitative Content Analysis according to Mayring [20] was chosen, as it is particularly suitable for enabling the development of new theories and categories. While this methodology is commonly used in social, healthcare and psychological research, it is less prevalent in dentistry. 

## 3. Results

During the survey period, a total of 257 accesses were documented. Of these, 109 individuals began the survey, with 47.7% (*n* = 52) completing the questionnaire. Among the 52 fully completed questionnaires, 18 (34.6%) were from paper questionnaires distributed during the BKJPP conference, and 34 (65.4%) were from online questionnaires. This corresponds to a response rate of 10.9% among approximately 1000 specialized physicians organized within BKJPP at the time of the survey, with a completion rate of 5.2% for fully filled questionnaires. One closed-ended question had to be retrospectively excluded from the study due to unanalyzable responses, leaving 38 closed-ended questions.

### 3.1. Sociodemographic Data

**Figure 1:**
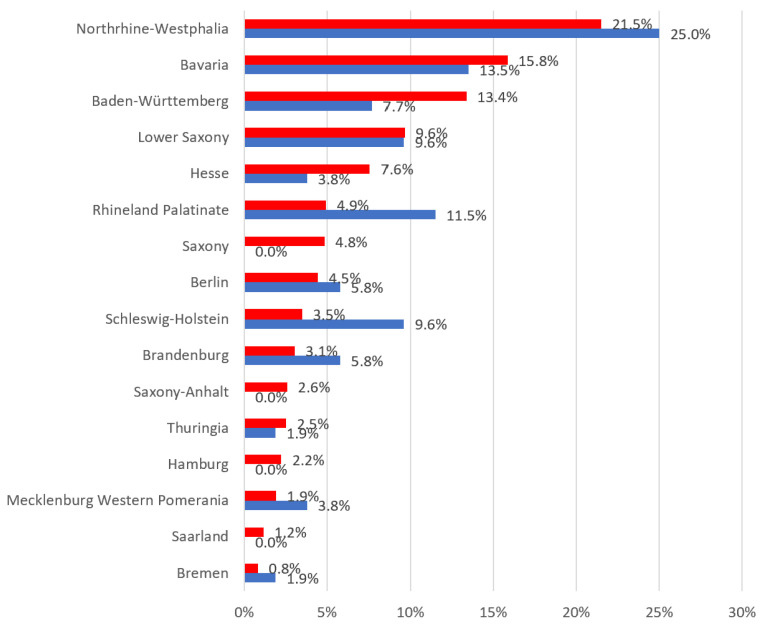
Distribution of respondents (blue) compared to the German population (red) by federal states.

As shown in Table 1, the majority of participants were between 55 and 65 years old (57.7%; *n* = 30), had over 20 years of professional experience (61.5%; *n* = 32) and were predominantly female (73.1%; *n* = 38), as expected based on the current statistics regarding German psychiatrists (avg. age in 2022: 54.1 years.; avg. female proportion in 2022: 66.1% [21]). Most participants were in practices licensed by statutory health insurance companies (75%; *n* = 39) and worked with a multidisciplinary team (86.5%; *n* = 45). The practices and clinics were mostly located in urban areas (80.8%; *n* = 42). Nearly all participants (98.1%; *n* = 51) were specialized physicians in child and adolescent psychiatry and psychotherapy. The majority (84.6%; *n* = 44) reported that less than ten percent of patients in their practice or clinic were privately insured. Half of the participants (46.2%; *n* = 24) estimated that approximately five percent of patients were privately insured. Figure 1 illustrates that data from four federal states were missing (Hamburg, Saarland, Saxony-Anhalt and Saxony), while the remaining states reflected a return rate that approximately mirrored the population distribution.

Table 1 also depicts the participants’ descriptions of their practices’ wheelchair accessibility. Half of the participating CAPPS indicated that their practices were not wheelchair-accessible (50%; *n* = 26). Additionally, one-third of participants stated that their practice rooms were not wheelchair-accessible-furnished at all (32.7%; *n* = 17). Nevertheless, 78.8% of participants (*n* = 41) claimed their practices were geared towards serving individuals with disabilities.

**Figure 2:**
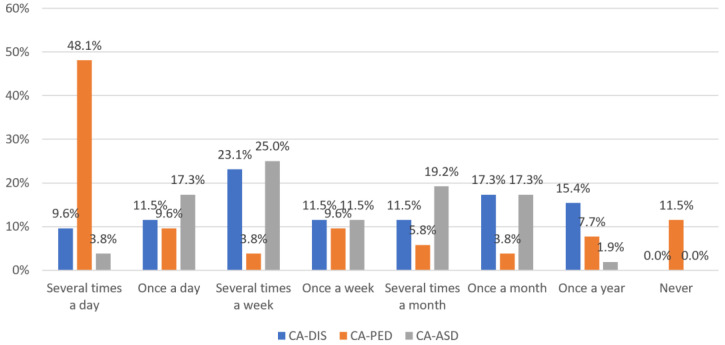
Treatment frequency of different patient groups in the sample. Abbreviations: CA-DIS = Child and Adolescents with disabilities; CA-PED = Children and Adolescents with psychoemotional behavioral disorders; CA-ASD = Children and Adolescents with autism spectrum disorders.

The frequency of treating various patient groups is evident in Figure 2. Approximately one-fifth of participants reported treating one or more children and adolescents with disabilities daily (21.1%; *n* = 11). In contrast, about one-third of participants (32.7%; *n* = 17) stated they treated children and adolescents with disabilities about once a month or less frequently. A majority of participating CAPPS treated patients with psychoemotional behavior disorders daily (57.7%; *n* = 30), with nearly half treating them multiple times (48.1%; *n* = 25). Approximately one-fifth (21.1%; *n* = 11) treated at least one patient with autism spectrum disorder daily.

### 3.2. Relevance of Dental and Oral Health in Child and Adolescent Psychiatric Therapy

**Figure 3:**
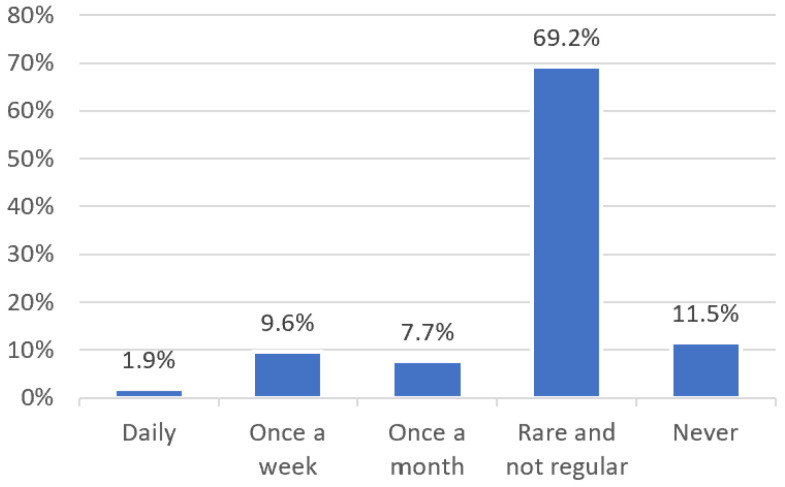
Frequency distribution of dental and oral health issues in the context of child and adolescent psychiatric treatment.

Around one-fifth of participants reported that dental and oral health was discussed in conversations and examinations at least once a month (19.2%; *n* = 10; see Figure 3). Similarly, 11.5% of participants (*n* = 6) stated that dental and oral health was “never” discussed. Dental topics were mainly discussed concerning dental treatment fears or when an attestation was needed for dental treatment or maxillofacial surgery under general anesthesia, primarily addressing patient concerns within the psychiatric context. Rarely were topics such as “toothaches” or “dental aesthetics” mentioned (see Table 2).

Overall, 16 participants (30.8%) provided additional information, such as details about when or to what extent dental and oral health topics were discussed. Due to the small sample size and heterogeneous, highly individual responses, a qualitative content analysis according to Mayring [20] seemed unfeasible at this point considering all the quality criteria. However, three categories could be tentatively derived from the analysis of 17 comments to support respective categories and enable initial hypothesis and theory formation.

1. **Disorder-Specific Indication:** “*Primarily in patients with depressive and/or psychotic disorders due to neglect of personal hygiene*”
2. **Patient Concerns:** “*I don’t specifically inquire about dental health. However, if problems are reported to me, I address them*” and “*Children and adolescents with dental phobia often seek certification for dental surgery under general anesthesia*”
3. **Lack of Consideration of Dental and Oral Health in Daily Practice:** “*The topic is primarily discussed with pediatricians, less frequently in CAPPS practices, despite frequent fears and psychosomatic symptoms*” and “*We have not considered this aspect frequently enough.*”

These three inductively derived categories seem to align with previously quantitatively queried topics on dental and oral health. Only 9.6% of participants (*n* = 5) reported being familiar with and possibly coding ICD-10 diagnoses related to dental and oral health (K00–K14). If coded, the most common diagnosis was K02—Dental Caries. Two participants (3.8%) reported coding this diagnosis more than once a week, and three others (5.8%) reported coding it “multiple times”. One person referred patients in the past to a colleague in oral, maxillofacial surgery (1.9%), a private dentist (1.9%) or a university dental clinic (1.9%).

### 3.3. Explanatory Factors for Poor Oral Health in Children and Adolescents with Special Support Needs

The participants had the opportunity to comment on possible reasons for the poorer dental and oral health of children, adolescents and young adults with disabilities, psycho-emotional behavior disorders or autism spectrum disorder. Of the participants, 42 (80.8%) took advantage of this opportunity. A total of 121 responses were collected and summarized for content redundancy. A qualitative content analysis based on Mayring [20] was conducted, aided by a previously created coding guide. From the analysis of the 121 responses, four categories were inductively generated to express explanatory factors.

1. **Behavioral Causes:** A lack of cooperation and compliance (21x), inadequate dental care (10x), unhealthy eating and drinking habits (7x), a lack of understanding or insight into dental hygiene importance (6x), a lack of self-care (5x), a lack of independence (2x), a lack of knowledge (2x), low perseverance (1x), general deficits in personal hygiene (1x), unpleasant sensations during toothbrushing (1x)
2. **Illness-Related Causes:** Dental phobia (11x), altered motor skills (e.g., only drinking from a bottle, poor chewing) or disease-related malocclusion (8x), motor difficulties (5x), altered saliva flow (3x), genetic predispositions (2x), reduced pain tolerance (2x), a lack of body awareness (1x), bruxism (1x)
3. **High Psychosocial Burden on the Family System:** Overwhelming of caregivers (5x), a lack of healthcare by caregivers (4x), low priority given to dental health (4x), dental visits perceived as too burdensome (3x), a lack of care and guidance by caregivers (2x), financial burdens (1x) Insufficient Support and Care from the Support System: Inadequate support for caregivers (3x), dental co-treatment or assessment often neglected (1x), dental health often given low priority (1x), dental visits deemed too burdensome (1x)
4. **Communication Barriers:** A lack of or very difficult patient-side communication (e.g., language) (7x)

### 3.4. Desires and Suggestions for Improving Dental Patient Care and Collaboration

The participants had the opportunity to express wishes or suggestions for improving the care and treatment of patients concerning dental and oral health. Of the participants, 22 (42.3%) took advantage of this opportunity. However, some responses overlapped with previous answers (e.g., response: “see above”), or it was mentioned that previously mentioned deficiencies needed to be addressed. After reviewing the responses, 17 new insights and concrete suggestions for improving the care problem from child and adolescent psychiatric perspectives were identified. Using qualitative content analysis according to Mayring [20] and a previously created coding guide, two categories were inductively generated:

1. **Expand Cooperation and Networks:** Share more information about specialized treatment options or specialized dentists (3x), Enhance region-specific networking on dental health (2x), Enable Research and Education: Provide specific education for CAPPS (2x), Offer specific training for dentists (2x), Expand scientific engagement at the interface of child and adolescent psychiatry and dentistry (2x)
2. **Structural Changes in Daily Practice:** Offer larger time slots where possible (5x), Acquire noiseless drills and low-stimulus treatment rooms (1x)

## 4. Discussion

This study provides initial insights into how Child and Adolescent Psychiatrists (CAPPS) in Germany assess the importance of dental and oral health issues in their own child and adolescent psychiatric therapy. Additionally, this study examines how this medical specialty deals with dental and oral health issues in children and adolescents with disabilities, psychoemotional behavior disorders, and autism spectrum disorder, thus representing a novel contribution to the scientific establishment of this interface.

In the overarching analysis of quantitative and qualitative data, it becomes evident that, from CAPPS, there are few fundamental differences regarding framework conditions, working methods and personal attitudes depending on the underlying type of daily-life restriction (physical/mental disability, psychoemotional behavior disorder, autism spectrum disorder). Regarding the importance of dental and oral health issues, there are no significant differences observed among the three target groups in the overarching analysis, prompting us to dissolve the clear distinction between these subgroups and subsume them under the term “Children and Adolescents with Special Support Needs”, abbreviated as CA-SN hereafter. The subsumption of various vulnerable patient groups with disabilities or other impairments, such as physical or mental disabilities, psychological behavioral disorders or developmental disorders, like autism spectrum disorder, under the titles “special needs”, “special support needs” or “special healthcare needs” is quite common in dentistry, and the terms are also used synonymously with each other to some extent [11,12,13,14,15,16,22,23,24,25]. 

In explaining the genesis of the poorer dental and oral health of CA-SN, CAPPS predominantly refer to a bio-psycho-social model, which is already standard in most “talking medicine”. From a CAPPS perspective, the main reasons for the poor dental status of CA-SN are primarily patient- and disease-related factors; additionally, factors arising from the high psychosocial burden on the family system, inadequate support from the help system and communication barriers also contribute. 

Causes for the poorer oral health status in CA-SN, from an international dental perspective, include irregular dental hygiene, often stereotypical unhealthy eating habits, decreased cooperation, a lack of dentists trained in special care dentistry, the absence of barrier-free clinics and the lack of specialized treatment concepts [22,23,24,26,27]. These dental perspectives align with the data gathered from the CAPPS and the national dental perspectives, mentioned in the introduction. 

The survey illustrates that there is indeed an overlap between CAPPS and dentists in the explanatory approaches to poorer dental and oral health in CA-SN, referring to, for example, behavioral causes, illness-related causes and communication barriers [22,24,26,27]. Furthermore, the collected data cautiously suggest that CAPPS, in addition to the poorer dental and oral health status of CA-SN, tend to identify familial or psychosocial factors, such as the overwhelming of caregivers or the low priority given to dental health, while the dental profession is more likely to point out structural deficits, such as the lack of continuing education and training, the lack of research or the lack of financial incentives [22,23,24,26,27]. 

It is imperative to pay particular attention to the oral health of these vulnerable groups, as internationally, CA-SN have a higher risk of developing dentogenic diseases (e.g., caries), which are often treated belatedly due to the reasons mentioned above, leading to the increased severity of the diseases [23,24,26,28,29,30]. Consequently, CA-SN often require more invasive procedures (e.g., general anesthesia) [25]. From the perspective of dentistry, there is an increasing urgent need for preventive and treatment-specific approaches and concepts for this vulnerable group [26]. A core element of this preventive work lies in the regular attendance of check-up appointments to timely detect and treat dentogenic diseases [28]. In a sample from Saudi Arabia, it was shown (considering the significant differences in various healthcare systems) that only 55.8% of parents of autistic children regularly bring their children to the dentist (as recommended, twice a year), and one-third of parents believe that a dental visit is only necessary when CA-SN experience dental pain [31]. At this point, the necessity of low-threshold psychoeducation and counseling becomes evident.

On an international level, the dental profession emphasizes the necessity of establishing larger networks and collaborations with psychotherapists, psychiatrists and dentists to facilitate low-threshold and early access to adequate dental care [23,26,28]. In the Saudi Arabian sample, it was shown that almost 20% of parents would contact their (trusted) general practitioner if their child experienced dental pain [31]. This could underscore the importance of a trusting and long-term doctor–patient–family relationship, as family members may turn to familiar and trusted doctors rather than directly to the appropriate interface. The timely referral and sharing of information by possibly existing familiar physicians regarding specialized dentists and the importance of dental and oral health topics could contribute to CA-SN gaining earlier access to dental care and thereby ensuring that dental prevention and treatment take place, thus reducing the risk of early-onset dentogenic diseases going undetected or untreated.

CAPPS regularly treat these patients and, over the sometimes-long treatment periods, have often established a good working alliance with the patients and caregivers. Thus, through their relationship-based work, this medical specialty would have the best conditions to make recommendations (e.g., specialized dental specialists in the region) and thereby fundamentally improve the quality of care in this regard. For this purpose, it seems sensible and necessary to routinely include questions about dental and oral health in the basic medical history. It is not about CAPPS providing dental care but being organized in regional networks that can provide reference points. This also includes setting logistical and financial incentives for such multiprofessional therapy planning, discussions and treatments [28]. This aligns with the demands and desires of surveyed CAPPS to engage the scientific exchange, establish regional networks and offer further education on oral health-related topics.

Long-term, this could lead to the development of multiprofessional and interdisciplinary preventive treatment concepts, thus enabling the early detection and treatment of dentogenic diseases to reduce the severity of such diseases and the frequency of invasive procedures under general anesthesia [22,24,26]. This would also contribute to the adequate utilization of existing (financial) resources. 

The importance of the seemingly niche topic of dental and oral health is measurable primarily by the number of coded diagnoses. Only 9.6% of participants reported knowing the relevant ICD-10 diagnoses, with only 3.8% using them regularly, consistent with the analysis of the corresponding routine data. According to the report on the 100 most frequent ICD-10 codes and short texts of the Association of Statutory Health Insurance Physicians North Rhine from the second quarter of 2022, fewer than 0.1% of psychotherapeutically active physicians (in the morbidity statistics, the term “psychotherapeutically active physicians” includes the specialist groups “child and adolescent psychiatry, psychosomatics and psychotherapy”, “psychiatry and psychotherapy” and “psychosomatic medicine and psychotherapy”) assign a diagnosis related to mouth or dental status [32]. These regional data can be validated by the nationwide analysis of statutory health insurance billing datasets provided by the Zentralinstitut (ZI). In the analysis of nationwide billing data from GKV-insured individuals (0–44 years) from 2019 (ZI-data sets), the prevalence of diagnoses related to oral health in individuals with mental illness, as well as the same-aged general population, was found to be very low [33]. The rare assignment of diagnoses related to dental and oral health suggests that claims data for oral health-related diagnoses made by physicians are not suitable for realistically depicting the the prevalence of dental and oral health of a specific population or sample [33]. The reasons for the infrequent coding of dental and oral health-related diagnoses are diverse and require further investigation. A look into the training regulations or into the training plan of the federal government and the various states also shows that, currently, they do not include topics related to dental and oral health [34,35].

It is important to recognize that CA-SN face particular challenges regarding their oral health not only due to their neurologically–psychologically–developmentally related differences but also due to barriers to dental care and specific treatment needs. A respectful and secure approach in clinical facilities tailored to the needs of this vulnerable group, as well as well-trained-dentists, functional regional networks and continuously evolving research, are indispensable for addressing these challenges. 

Therefore, it appears promising if CAPPS are made aware of the necessity and importance of this issue. However, this requires short, practice-oriented training, as the topic is neither firmly anchored in the training regulations nor in the training plan. It would also be sensible and, in our view, necessary to include the assessment or monitoring of dental status in the child and adolescent psychiatric intake examination and, if necessary, to refer to specialized dentists. In newer textbooks, the assessment of dental status is mentioned at least briefly [36]. The results of this study underline the need for the development of a short and practice-oriented core curriculum with content on dental and oral health.

## 5. Strengths and Limitations of the Methodology, Implementation and Data Analysis

The strengths are certainly rooted in the methodological approach. We opted for Mayring’s content analysis [20], as it appears suitable for deriving new theories from real-life experiences and practice. In addition to the three universally accepted criteria of validity, reliability and objectivity, we also included the criteria of transparency, intersubjectivity and scope. The methodological process within the qualitative content analysis was continuously reflected upon using the six criteria. It was important to describe the procedure in detail and critically (transparency), to clearly separate results and discussion (intersubjectivity) and to achieve theoretical saturation to the greatest extent possible (scope). Despite the low absolute participation numbers, we largely consider the criterion of scope to be fulfilled. The response rate of the survey is comparable, in percentage terms, to a survey conducted among members of the German Society of Pediatric Dentistry (DGKiZ) (based on the response rate: 11.1% in DGKiZ and 10.9% in BKJPP; based on completeness: 5.3% in DGKiZ and 5.2% in BKJPP; see [11]). Another limitation is that the present survey was only conducted among members of the BKJPP. The nationwide survey results presented here can, in our opinion, initially be considered as a first milestone with representative characteristics. This conclusion from the representative survey is supported by sociodemographic data such as the gender ratio and regional percentage distribution (Figure 1). Due to the numerous qualitative responses (free-text answers), it can be assumed that the respondents had a high interest in the topic.

## 6. Conclusions

CA-SN face significant barriers and often receive inferior dental care, resulting in poorer overall oral health. To further improve the dental and oral health of CA-SN and to develop new concepts, ongoing research is needed. Currently, there are no data available from the perspective of the affected patients, nor are there data from the perspective of child and adolescent psychotherapists. 

The in-depth examination of dental and oral health issues from a child and adolescent psychiatric perspective is certainly new, and there is a lack of established foundations for everyday practice. In this context, the child and adolescent psychiatric perspective clearly emphasizes the need for widespread cooperation and the establishment of interfaces and interdisciplinary care concepts, as can already be found in regional beacon models. This also reflects the desire among participating child and adolescent psychiatric professionals for more regional networking and information about specialized treatment options. 

The qualitative analysis shows that the topic of oral and dental health often becomes part of psychiatric therapy when children and adolescents specifically bring it up. This primarily concerns fears of dental treatment or the issuance of medical certificates for wisdom tooth surgery under general anesthesia. Moreover, the topic of dental and oral health often finds no or only rare entry into child and adolescent psychiatric treatment. 

## Figures and Tables

**Table 1:** Characteristics of the study participants (members of the Professional Association for Child and Adolescent Psychiatry, Psychosomatics and Psychotherapy in Germany). Abbreviations: CAPP = Child and Adolescent Psychiatrist.

Description of the Sample	Frequencies = 52	
**Gender**		
male	14	26.9%
female	38	73.1%
**Age**		
<35 years old	-	-
35–44 years old	5	9.6%
45–54 years old	11	21.2%
55–64 years old	30	57.7%
>65 years old	6	11.5%
**Professional experience (in years)**		
less than 5 years	1	1.9%
5–10 years	2	3.8%
11–15 years	7	13.5%
16–20 years	10	19.2%
more than 20 years	32	61.5%
**Current field of activity**		
established in own practice	39	75%
employed in a hospital as a CAPP	4	7.7%
employed as a CAPP in a university clinic	3	5.8%
employed in a care center (MVZ) as a CAPP	3	5.8%
employed as a CAPP in a practice	2	3.8%
employed as an assistant doctor in a practice	1	1.9%
**Local–regional situation**		
village location	10	19.2%
urban location	42	80.8%
**Type of insurance of the patients**		
statutorily insured		90%–95%
privately insured		5%–10%
**Wheelchair-accessible practice**		
rooms are wheelchair-accessible	35	67.3%
rooms are wheelchair-accessible-furnished	26	50%

**Table 2:** Frequency of dental and oral health issues in child and adolescent psychiatric therapy and treatment (multiple answers possible). Abbreviations: CA-DIS = Children and Adolescents with disabilities; CA-PED = Children and Adolescents with psychoemotional behavioral disorders; CA-ASD = Children and Adolescents with autism spectrum disorders.

	Dental Anxiety	Teeth and Dental Health	Teeth and Nutrition	Toothache	Teeth and Self-Harm	Dental Aesthetics	Others
CA-DIS	80.8% (42)	53.6% (28)	40.4% (21)	26.9% (14)	26.9% (14)	15.4% (8)	7.7% (4)
CA-PED	71.2% (37)	46.2% (24)	38.5% (20)	25.0% (13)	17.3% (9)	21.2% (11)	15.4% (8)
CA-ASS	82.7% (43)	52.0% (27)	30.8% (16)	23.1% (12)	21.2% (11)	11.5% (6)	7.7% (4)

## Data Availability

The data and the questionnaire presented in this study are available on request from the corresponding author (D.F.). The data are not publicly available due to privacy reasons.

## References

[1] Statistisches Bundesamt Behinderte Menschen: Schwerbehinderte Menschen am Jahresende. https://www.destatis.de/DE/Themen/Gesellschaft-Umwelt/Gesundheit/Behinderte-Menschen/Tabellen/geschlecht-behinderung.html.

[2] Bundespsychotherapeutenkammer Fast 20 Prozent Erkranken an Einer Psychischen Störung: BPtK-Faktenblatt “Psychische Erkrankungen bei Kindern und Jugendlichen”. https://bptk.de/pressemitteilungen/fast-20-prozent-erkranken-an-einer-psychischen-stoerung.

[3] Ravens-Sieberer U., Otto C., Kriston L., Rothenberger A., Döpfner M., Herpertz-Dahlmann B., Barkmann C., Schön G., Hölling H., Schulte-Markwort M. (2015). The longitudinal BELLA study: Design, methods and first results on the course of mental health problems. Eur. Child Adolesc. Psych..

[4] Klipker K., Baumgarten F., Göbel K., Lampert T., Hölling H. (2018). Psychische Auffälligkeiten bei Kindern und Jugendlichen in Deutschland—Querschnittergebnisse aus KiGGS Welle 2 und Trends. J. Health Monit..

[5] Schmidt P., Egermann M., Sauerland C., Schulte A.G. (2021). Caries Experience of Adults with Intellectual Disability in the Western Part of Germany. J. Clin. Med..

[6] Schulte A.G., Schmidt P. (2021). Mundgesundheit bei Menschen mit Behinderung in Deutschland—Eine Literaturübersicht. Bundesgesundheitsblatt-Gesund..

[7] Schüler I.M., Dziwak M., Schmied K., Lehmann T., Heinrich-Weltzien R. (2019). Mundgesundheit von Kindern und Jugendlichen mit geistiger Behinderung und psychoemotionalen Störungen aus Niedersachsen und Thüringen. Gesundheitswesen.

[8] Wilson N.J., Lin Z., Villarosa A., George A. (2019). Oral health status and reported oral health problems in people with intellectual disability: A literature review. J. Intellect. Dev. Dis..

[9] Zucoloto M.L., Maroco J., Campos J.A.D.B. (2016). Impact of oral health on health-related quality of life: A cross-sectional study. BMC Oral Health.

[10] Heinrich-Weltzien R., Wagner A., Micheelis W. (2013). Fachwissen und subjektive Behandlung von Kindern mit Behinderungen: Eine Befragung der Thüringer Zahnärzteschaft. Oralprophylaxe Kinderzahnheilkd..

[11] Schmidt P., Reis D., Schulte A.G., Fricke O. (2022). Self-Assessment of Knowledge on the Treatment of Children and Adolescents with Special Care Needs: Results of a Survey amongst German Dentists with Key Expertise in Paediatric Dentistry. J. Pers. Med..

[12] Casamassimo P.S., Seale N.S., Ruehs K. (2004). General Dentists’ Perceptions of Educational and Treatment Issues Affecting Access to Care for Children with Special Health Care Needs. J. Dent. Educ..

[13] Dao L.P., Zwetchkenbaum S., Inglehart M.R. (2005). General Dentists and Special Needs Patients: Does Dental Education Matter?. J. Dent. Educ..

[14] Schmidt P., Egermann M., Ehlers J.P., Schulte A.G. (2023). A five-year cohort study on German dental students: Self-assessment in regard to previous experience and attitude toward patients with different types of disability. Spec. Care Dentist..

[15] Reis D., Fricke O., Schulte A.G., Schmidt P. (2022). Is examining children and adolescents with autism spectrum disorders a challenge?-Measurement of Stress Appraisal (SAM) in German dentists with key expertise in paediatric dentistry. PLoS ONE.

[16] Weil T.N., Bagramian R.A., Inglehart M.R. (2011). Treating patients with autism spectrum disorder--SCDA members’ attitudes and behavior. Spec. Care Dentist..

[17] Schüler I.M., Bock B., Heinrich-Weltzien R., Bekes K., Rudovsky M., Filz C., Ligges C. (2017). Status and perception of oral health in 6-17-year-old psychiatric inpatients-randomized controlled trial. Clin. Oral Investig..

[18] Machetanz E. (1977). Probleme zahnärztlicher Behandlung bei geistig und seelisch gestörten Kindern unter dem Aspekt der Jugendpsychiatrie. Zahnärztl. Mitt..

[19] Döring N., Bortz J., Pöschl S., Werner C.S., Schermelleh-Engel K., Gerhard C., Gäde J.C. (2016). Forschungsmethoden und Evaluation in den Sozial- und Humanwissenschaften.

[20] Mayring P. (2022). Qualitative Inhaltsanalyse: Grundlagen und Techniken.

[21] Kassenärztliche Bundesvereinigung Gesundheitsdaten: Vertragsärztliche Versorgung. https://gesundheitsdaten.kbv.de/cms/html/16392.php.

[22] Jones J., Roberts E., Cockrell D., Higgins D., Sharma D. (2024). Barriers to Oral Health Care for Autistic Individuals-A Scoping Review. Healthcare.

[23] Sami W., Ahmad M.S., Shaik R.A., Miraj M., Ahmad S., Molla M.H. (2023). Oral Health Statuses of Children and Young Adults with Autism Spectrum Disorder: An Umbrella Review. J. Clin. Med..

[24] Hasell S., Hussain A., Da Silva K. (2022). The Oral Health Status and Treatment Needs of Pediatric Patients Living with Autism Spectrum Disorder: A Retrospective Study. Dent. J..

[25] Biasotto M., Poropat A., Porrelli D., Ottaviani G., Rupel K., Preda M.T.B., Di Lenarda R., Gobbo M. (2024). Dental Treatment in Special Needs Patients and Uncooperative Young Children: A Retrospective Study. Medicina.

[26] Bossù M., Trottini M., Corridore D., Di Giorgio G., Sfasciotti G.L., Palaia G., Ottolenghi L., Polimeni A., Di Carlo S. (2020). Oral Health Status of Children with Autism in Central Italy. Appl. Sci..

[27] Pimentel Júnior N.S., de Barros S.G., de Jesus Filho E., Vianna M.I.P., Santos C.M.L., Cangussu M.C.T. (2024). Oral health-care practices and dental assistance management strategies for people with autism spectrum disorder: An integrative literature review. Autism.

[28] Corridore D., Zumbo G., Corvino I., Guaragna M., Bossù M., Polimeni A., Vozza I. (2020). Prevalence of oral disease and treatment types proposed to children affected by Autistic Spectrum Disorder in Pediatric Dentistry: A Systematic Review. Clin. Ter..

[29] Uliana J.C., Del’ Agnese C.C., Antoniazzi R.P., Kantorski K.Z. (2024). Autistic individuals have worse oral status than neurotypical controls: A systematic review and meta-analysis of observational studies. Clin. Oral Investig..

[30] Verma A., Priyank H., Viswanath B., Bhagat J.K., Purbay S., V M., Shivakumar S. (2022). Assessment of Parental Perceptions of Socio-Psychological Factors, Unmet Dental Needs, and Barriers to Utilise Oral Health Care in Autistic Children. Cureus.

[31] Alqahtani A.S., Gufran K., Alsakr A., Alnufaiy B., Al Ghwainem A., Bin Khames Y.M., Althani R.A., Almuthaybiri S.M. (2023). Oral Healthcare Practices and Awareness among the Parents of Autism Spectrum Disorder Children: A Multi-Center Study. Children.

[32] Kassenärztliche Vereinigung Nordrhein Die 100 häufigsten ICD-10-Schlüssel und Kurztexte (nach Fachgruppen): 2. Quartal 2022. https://www.kvno.de/fileadmin/shared/pdf/online/verordnungen/morbiditaetsstatistik/100icd_22-2.pdf.

[33] Schmidt P., Reis D., Schulte A.G., Fricke O. (2022). Zahnmedizinische Diagnoseprävalenzen bei Kindern, Jugendlichen und jungen Erwachsenen mit psychischen Störungen im Vergleich zu Gesunden-Analyse und Abschätzung kassenärztlicher Abrechnungsdaten (2019). Psychother. Psychosom. Med. Psychol..

[34] Bundesärztekammer (Muster-)Fachlich Empfohlene Weiterbildungspläne: Fachlich Empfohlener Weiterbildungsplan für den/die Facharzt für Kinder- und Jugendpsychiatrie und -Psychotherapie. https://www.bundesaerztekammer.de/fileadmin/user_upload/BAEK/Themen/Aus-Fort-Weiterbildung/Weiterbildung/FEWP/FA_SP-WB/20201112_13_FEWP_KJPP.pdf.

[35] Ärztekammer Westfalen-Lippe Weiterbildungsordnung der Ärztekammer Westfalen-Lippe vom 02. April 2022 inkl. Richtlinien über den Inhalt der Weiterbildung. https://www.aekwl.de/fileadmin/user_upload/aekwl/weiterbildung/Aenderungsfassung_April_2022_-_WO_AEKWL_01.07.2023.pdf.

[36] Izat Y., Fegert J., Resch F., Plener P., Kaess M., Döpfner M., Konrad K., Legenbauer T. (2019). Körperliche Diagnostik in der Kinder- und Jugendpsychiatrie. Psychiatrie und Psychotherapie des Kindes- und Jugendalters.

